# Clinical and electrophysiological characteristics of ventricular arrhythmias arising from pulmonary cusps

**DOI:** 10.1002/joa3.12347

**Published:** 2020-05-08

**Authors:** Vickram V. Rangaswamy, Sachin Yalagudri, Daljeet K. Saggu, Muthiah Subramanian, Chennapragadha Sridevi, Calambur Narasimhan

**Affiliations:** ^1^ Department of Cardiology AIG Hospitals Gachibowli India

**Keywords:** RVOT PVC, supravalvular PVC, VT

## Abstract

**Introduction:**

Ventricular arrhythmias (VAs) have been successfully ablated from the pulmonary sinus cusps establishing pulmonary artery (PA) as a distinct site of arrhythmic foci. The aim of the present study was to determine the clinical presentation, electrocardiographic, and ablation characteristics of PA‐VAs.

**Methods:**

Thirty consecutive patients with right ventricular outflow tract (RVOT)‐type VAs were included in this retrospective study. Three‐dimensional electroanatomic mapping was performed in all patients. Mapping was performed initially in RVOT, and later within the PA. Mapping was performed in the PA if there was no early activation, unsatisfactory pace‐map, or ablation in RVOT were unsuccessful. All PA‐VAs were mapped and ablated by looping the catheter in a reverse U fashion.

**Results:**

Among 30 patients, 8 (26.6%) patients VAs were successfully ablated within PA. Electrocardiography (ECG) revealed that the QRS duration was significantly wider in the PA‐VAs group compared to the RVOT‐VAs group (155 ± 14.14 vs 142.40 ± 8.12 ms, *P* < .01). Mapping by reversed U method of PA‐VAs revealed earlier activation (55 ± 9.66 vs 12.00 ± 8.61 ms, *P* < .01) in PA compared to RVOT. An isolated discrete prepotential was present at the successful site in 50% (n = 4).

**Conclusion:**

Pulmonary artery‐VAs are an important subset of VA originating from the outflow tract. They have a wider baseline QRS duration compared to RVOT‐VAs. Presence of a prepotential aids in the identification of a successful ablation site. Mapping utilizing the reversed U method can help in localization and successful ablation of PA‐VAs.

## Graphical abstract

Pulmonary artery is an important site of ventricular arrhythmic foci. Presence of an isolated prepotential in pulmonary cusp helps to identify the successful ablation site. We found mapping utilizing the reversed U method can help in localization and ablation of pulmonary artery derived ventricular arrhythmia.

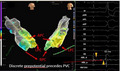

## 1 INTRODUCTION

Idiopathic ventricular arrhythmias (VAs) account for approximately 10% of patients referred for VA evaluation.^1^, ^2^, ^3^, ^4^ Majority of these VAs originate from the outflow tract. The success rate of catheter ablation of these arrhythmias is approximately 80%–95%.^5^ Ablation of outflow tract VAs originating from the pulmonary artery (PA) was first described by Timmermans et al^6^. Few studies have demonstrated successful ablation of VAs above the pulmonary sinus cusp (PSC) establishing the PA as a distinct site of right ventricular outflow tract (RVOT)‐VAs.^7^, ^8^, ^9^ However, there is no uniform approach or ablation strategy for this subset of VAs. The objectives of this study were to describe the clinical presentation, electrocardiographic, and ablation characteristics of PA‐VAs in comparison with RVOT‐VA.

## 2 METHODS

### 2.1 Study population

A total of 45 patients with left bundle branch block (LBBB) morphology and an inferior‐axis VAs underwent radiofrequency catheter ablation (RFCA) between January 2017 and April 2019. The indications for catheter ablation included symptomatic VAs with a PVC (premature ventricular complex) burden >10%, persistent symptoms despite drug therapy, left ventricular systolic dysfunction (LVD—defined as left ventricular ejection fraction [LVEF] less than 50%), and sustained ventricular tachycardia (VT). Cardiac magnetic resonance (CMR) and coronary angiography (CAG) was performed in patients with LVD to rule out secondary etiologies.

### 2.2 Electrocardiographic analysis

A 12‐lead electrocardiography (ECG) was recorded during the VAs. The following characteristics were analyzed: (a) QRS duration, (b) precordial R‐wave transition (earliest lead with R/S ratio >1), (c) R‐wave morphology in Lead I, (d) R‐wave amplitude and morphology in leads II, III, and aVF, (e) Q‐wave amplitude in leads aVR and aVL.

### 2.3 Radiofrequency ablation

**FIGURE 1:**
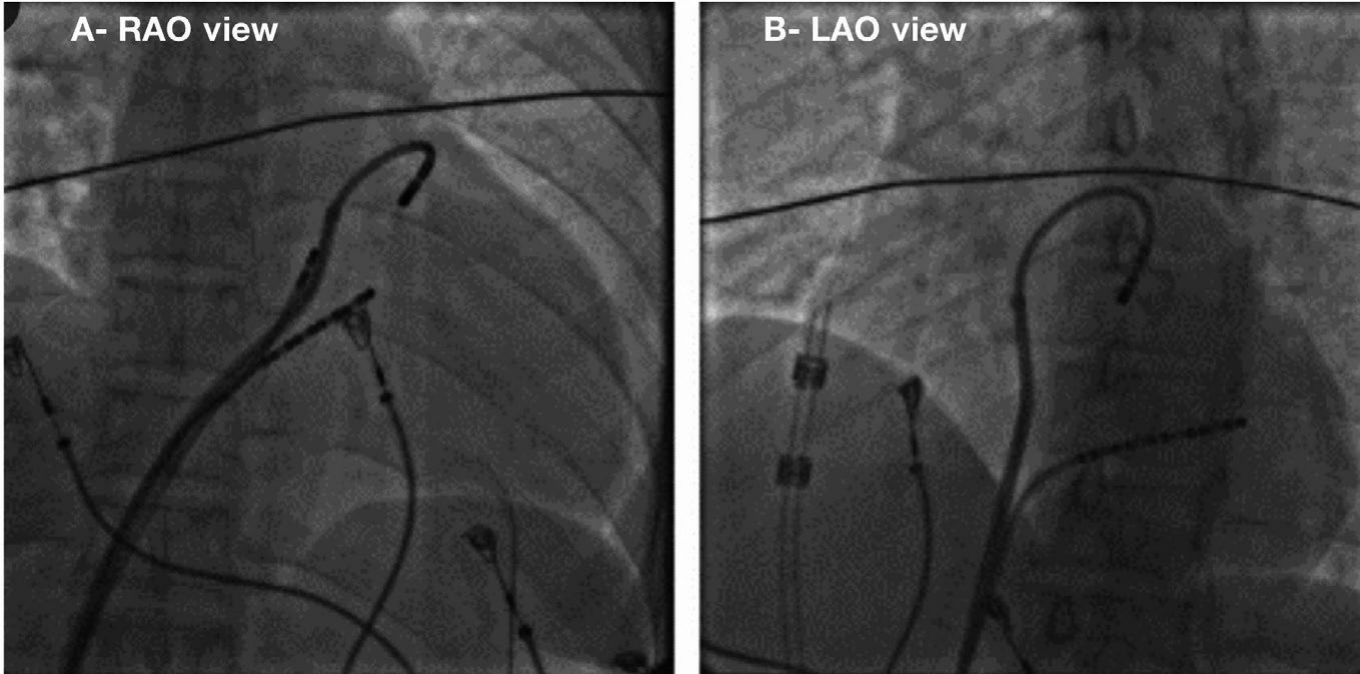
Right anterior oblique (RAO) and left anterior oblique view (LAO) radiographic view of mapping catheter in inverted U shape in anterior pulmonary cusp

All anti‐arrhythmic drugs were discontinued for at least five half‐lives before the procedure. Three‐dimensional (3D) electromagnetic mapping was performed using EnSite NavX™ (St. Jude Medical, Inc). A 7Fr irrigated tip ablation catheter with a 3.5‐mm tip was used for mapping and ablation. PA was mapped using a reversed U method in all patients. Preface® guiding sheath curve (8 F) (Biosense Webster Inc) was used to stabilize and manipulate the mapping catheter to access all three individual PSCs. The reverse U curve was made by curving the ablation catheter in the PA to position the tip into the PSC (Figure 1).

**FIGURE 2:**
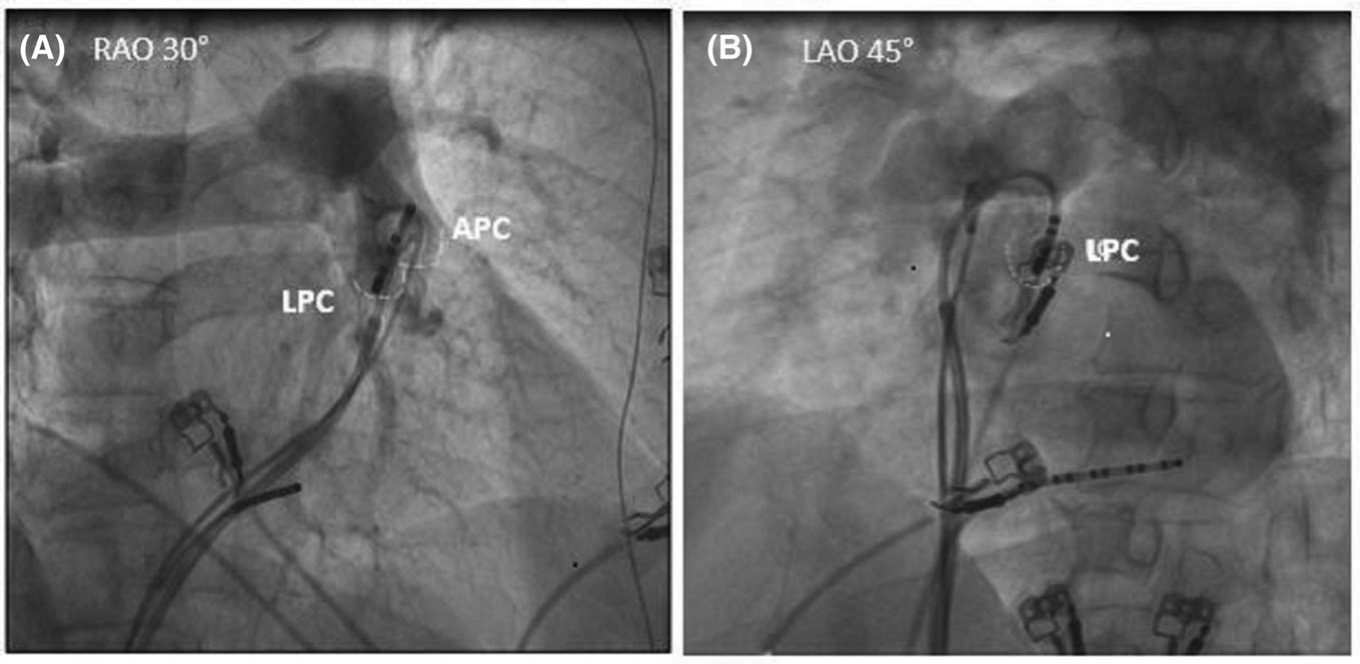
Pulmonary angiography in right anterior oblique view (RAO) and Left anterior oblique (LAO) view. APC, Anterior pulmonary cusp; LPC, Left pulmonary cusp

Activation mapping was used to identify the origin of VAs (Figure 2). If the clinical arrhythmia did not occur spontaneously or with programmed ventricular stimulation, intravenous isoproterenol (1‐4 μg min) was administered to provoke the arrhythmia. Initial activation mapping was performed in the RVOT region and if no early activation sites were identified, test burns were unsuccessful, or pace mapping was not good then the PSC was mapped. Activation time was based on the earliest bipolar signals and rapid down stroke of the unipolar signal correlating with the first sharp peak of the bipolar electrogram. In every patient, pace‐mapping was also performed. A pace‐map was considered “good” when score is ≥20.

**FIGURE 3:**
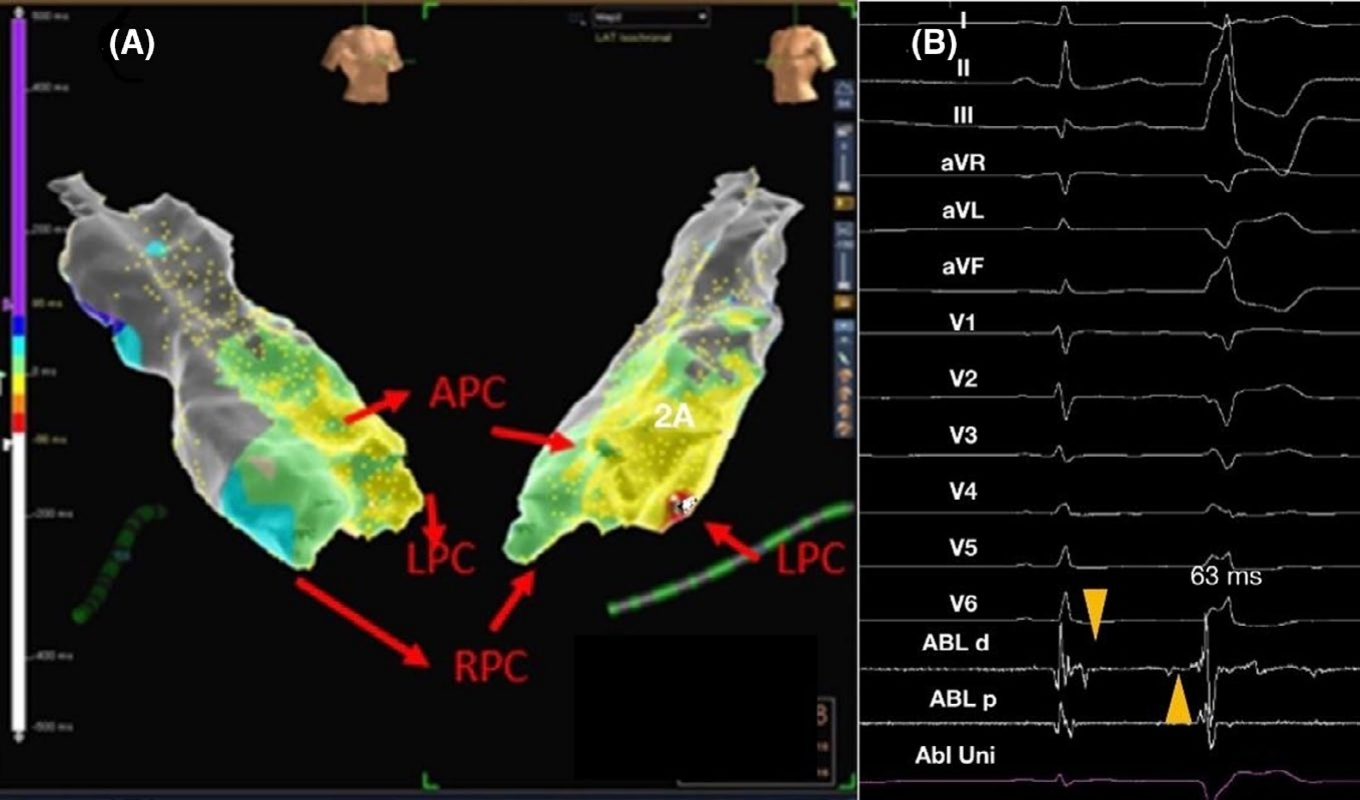
A, 3D activation mapping of pulmonary artery with cusps with relation of cusps in Right anterior oblique (RAO) and left anterior oblique view (LAO) view with earliest activation in the Left pulmonary cusp. B, Electrocardiogram with intracardiac signals from the map at the earliest site showing discrete isolated prepotential (with intervening isoelectric segment) (yellow arrow head). Discrete isolated prepotential precedes the premature ventricular complex QRS by 63 ms. In sinus rhythm, the prepotential follows the local bipolar electrogram. APC, Anterior pulmonary cusp; LPC, Left pulmonary cusp; RPC, Right pulmonary cusp

Ablation was performed at the site of earliest ventricular activation and/or good pace‐map. For PSC, catheter positions were evaluated in both right anterior oblique and left anterior oblique views. The position was confirmed by pulmonary angiogram done with a 6 French pig tail with a power injector (flow rate 30 cc/s at 1200 psi) (Figure 3). CAG was performed before RFCA to assess whether the left main coronary artery was within 5 mm of the ablation site. External irrigation with a flow rate of 17 ml/min, temperature cut off of 43°C, and a power range of 30–35 W was used. Acute success was defined as either absence of spontaneous or triggered clinical VAs at the end of the procedure. Patients were assessed for symptoms and twelve‐lead ECG/24‐hour Holter study was performed at the end of 3 months.

### 2.4 Statistical analysis

Continuous variables are expressed as mean ± SD, and categorical variables as numbers and percentages. Categorical variables were compared using chi‐square analysis or Fisher exact test. The parameters in different groups were compared by *t* test. A *P* value < .05 was considered significant.

## 3 RESULTS

### 3.1 Clinical characteristics

A total of 30 outflow tract—VAs originating from the RVOT and PA were included in the study. Among these, the sites of successful ablation were RVOT‐septum (n = 15), RVOT‐free wall (n = 7), and PA (n = 8). The comparison of baseline characteristics of PA‐VAs with RVOT group is shown in Table 1. Their mean age was 39.25 ± 10.16 years. The PVC burden was 15.25%. The mean LVEF of patients with PA‐VAs was 51.75 ± 7.28%.

**TABLE 1:** Comparision of baseline characteristics of PA‐VA AND RVOT VA

	PA‐VA (n = 8)	RVOT VA (n = 22)	*P*‐Value
Age, years	39.25 ± 10.16	51.82 ± 11.07	<.01
Female n (%)	6(75)	13(59.1)	.67
Symptoms n (%)			.99
1‐Palpitations	6 (75)	17 (77.3)	
2‐Syncope	2 (25)	5 (22.7)	
Arrhythmia n (%)			
PVC	3 (37.5)	15 (68.18)	
NSVT	4 (50)	0 (0)	.01
VT	1 (12.5)	7 (31.82)	
VA burden on 24‐h Holter Study	15.25 ± 5.09	14.45 ± 5.44	.72
LVEF (%)	51.75 ± 7.28	53.32 ± 11.74	.72

Abbreviations: LVEF, left ventricular ejection fraction; PA‐VA, pulmonary artery derived ventricular arrhythmia; RVOT‐VA, right ventricular outflow tract ventricular arrhythmia; VT, ventricular tachycardia.

Age of patients with PA‐VAs was significantly lower compared to RVOT‐VAs (39.25 ± 10.16 vs 51.82 ± 11.07 years, *P* < .01). Majority of PA‐VAs group had VT and NSVT at presentation compared to RVOT group (61.5% vs 38.5% *P* = .01). There was no significant difference in LVEF between these two groups (51.75 ± 7.28 vs 53.32 ± 11.7 *P* = .72). In PA‐VA group 25% (n = 2) had LVD. CAG and CMR were normal in patients with LVD. Medical therapy consisted of beta blockers, calcium channel blockers, amiodarone, or sotalol.

### 3.2 ECG characteristics

Electrocardiography characteristics of PA‐VAs and RVOT‐VAs are shown in Table 2. PVCs exhibited LBBB‐type morphology with inferior‐axis. Precordial QRS transition was noted in V3 in 25% (n = 2), V4 in 50% (n = 4), V5 in 12.5% (n = 1), and V6 in 12.5% (n = 1). The QRS duration was significantly wider in PA VA group compared to the RVOT group (155 ± 14.14 vs 142.40 ± 8.12 ms, *P* < .01). In addition, lead I positivity was more common in PA‐VAs (62.5% vs 18.18%, *P* = .02). The amplitude of R in II, III, aVF, and amplitude of Q wave in aVL and aVF did not differ significantly between the groups. In addition, lead III/II R wave ratio and aVL/aVR Q wave ratio were not different.

**TABLE 2:** Comparision of electrocardiographic characteristics of PA‐VA AND RVOT‐VA

	PA‐VA	RVOT VA	*P*‐Value
QRS duration,ms	155 ± 14.14	142.40 ± 8.12	<.01
Lead I (Positive) n (%)	5 (62.5)	4 (18.18)	.02
R wave amplitude in Lead II, mV	1.28 ± 0.70	2.04 ± 0.47	.06
R wave amplitude in Lead III, mV	1.32 ± 0.72	1.9 ± 0.57	.15
Q wave amplitude in Lead aVR, mV	7.2 ± 5.41	8.4 ± 2.6	.66
Q wave amplitude in Lead aVL, mV	0.83 ± 0.58	0.9 ± 0.31	.83
R wave amplitude in Lead aVF, mV	1.3 ± 0.69	1.64 ± 0.61	.38
Q wave amplitude ratio Lead aVL/aVR	0.12 ± 0.06	0.11 ± 0.03	.65
R wave amplitude ratio Lead III/II	1.03 ± 0.21	0.92 ± 0.07	.31
Notches II, III, aVF n (%)	2 (25)	0	.08

Abbreviations: PA‐VA, Pulmonary artery derived ventricular arrhythmia; RVOT‐VA, Right ventricular outflow tract ventricular arrhythmia.

### 3.3 Mapping and ablation characteristics of PA‐VA

The RVOT approach was successful in 22 (73.33%) patients but failed to eliminate/identify early site of arrhythmia in remaining eight patients. Among these eight patients, PA was mapped and successfully ablated in the PSC using a reversed U method. In PA, the successful ablation site was in the anterior pulmonary cusp (APC) in 4, left pulmonary cusp (LPC) in 3, right pulmonary cusp (APC) in 1 patient (Table 3). Spontaneous PVCs and non‐sustained VT occurred in five patients**.** In the remaining three patients, burst pacing with isoproterenol facilitation induced clinical arrhythmia.

**TABLE 3:** Electrophysiological features of PA‐VA

Pt. no	PSC	PM SCORE RVOT	PM SCORE‐AT PA	AT RVOT	AT PSC
1	Anterior	16	20	0	−38
2	Anterior	22	24	0	−52
3	Left	12	18	−12	−48
4	Right	10	21	−12	−58
5	Anterior	12	22	−10	−58
6	Left	18	22	−20	−96
7	Left	12	20	−22	−63
8	Anterior	12	24	−10	−30

Abbreviations: AT, activation time; PA, pulmonary artery; PA‐VAs, pulmonary artery derived ventricular arrhythmia; PM, Pace‐map; PSC, pulmonary sinus cusp; RVOT, right ventricular outflow tract.

**FIGURE 4:**
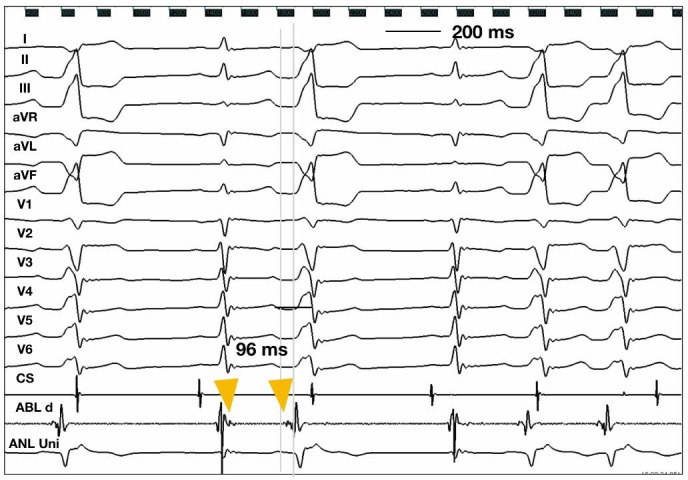
Electrogram showing bipolar recording with prepotential (without intervening isoelectric line) preceding QRS complex by 93 ms with simultaneous QS complex in PVC at the earliest activation site and reversal of this relationship in sinus rhythm. Prepotential, yellow arrow head

Patient's electrophysiological features are described in Tables 3 and 4. All PA‐VA patients underwent 3D mapping of RVOT and PA delineating individual cusps. In PA‐VA group, RVOT mapping showed bipolar electrogram preceding QRS onset by 12.00 ± 8.61 ms Ablation was initially attempted in four patients in RVOT, resulting in transient suppression of arrhythmia but it did not result in any change in QRS morphology. Subsequently RFCA was successfully performed in PA. In PA, ventricular activation at the successful ablation site was represented by two component bipolar electrogram in both sinus rhythm and clinical VAs in all patients. Analysis of bipolar signals in VAs revealed an initial sharp potential followed by a far field ventricular activation electrogram in all patients. This relationship was reversed during sinus rhythm. This sharp potential preceded the QRS by 55.37 ± 20 ms in PA. Analysis of systolic pre‐potentials revealed two types of prepotentials—first type was an isolated prepotential with an intervening isoelectric segment (Figure 3B) and the second type of prepotential was without an isoelectric line (Figure 4). Presence of an isolated prepotential helped identify the successful site in 50% (n = 4).

**TABLE 4:** Comparision of mapping parameters between PA‐VA AND RVOT‐VA

	RVOT	PA	*P* value
Activation time (ms)	12.00 ± 8.61	55.37 ± 20	<.01
Presence of prepotential at the earliest site	12.5%	100%	<.01
Unipolar QS electrogram	37.5%	62.5%	.333
Pace‐map score^a^	14.25 ± 4.06	21.37 ± 2.06	<.01
Radiofrequency Lesions	2.5 ± 1.30	2.87 ± 0.64	

Abbreviations: PA, pulmonary artery; PA‐VA, pulmonary artery derived ventricular arrhythmia; RVOT, right ventricular outflow tract; VA, ventricular arrhythmia.

^a^ Pace‐map score out of 24.

Pace‐mapping at the site of successful ablation was obtained in all patients. In PA‐VAs group 87.5% (n = 7) of patients had a good pace‐map. Pace‐map score in PA‐VAs group was better in PA compared to RVOT (14.25 ± 4.06 vs 21.37 ± 2.06, *P* < .01). Pace‐mapping in PA required higher output to capture the myocardium (10 mA) compared to RVOT‐VA. At successful sites, simultaneous unipolar QS morphology was recorded in 75% (n = 6) patients in PA. The mean procedure and fluoroscopy time were 135 ± 32 and 18 ± 8.6 minutes, respectively.

### 3.4 Follow‐up

Patients were followed up for a median of 13 (range 4‐36) months, and there was no recurrence of symptoms or clinical VA. No acute or long‐term procedural complications like coronary artery injury, pericardial effusion, pulmonary valve damage leading to pulmonary regurgitation or stenosis, occurred in any of the patients. All patients were off anti‐arrhythmic drugs at follow up. In addition, there was normalization of LV function in the two patients who initially presented with LVD.

## 4 DISCUSSION

The main finding of the study was that PA mapping should be considered when ablation fails to suppress VA in the RVOT. Clinical clues for PA‐VA include a younger age at presentation and absence of good sites/signals on mapping below the pulmonary cusp. In addition, the presence of a discrete prepotential can help identify the successful site of arrhythmia ablation.

### 4.1 Baseline clinical characteristics

The anatomical basis of PA‐VAs lies in the myocardial extensions in the PA above the cuspal and intercuspal region.^10^, ^11^ In this study, 26.66% of RVOT‐VAs originated in the PA. The wide variation in the reported frequency of PA‐VA is probably due to the earlier belief that myocardial signals terminate precisely at the annular level. The possible reason for this variation may be difficulty in identification of the PA fluroscopically or with 3D mapping. Although not used routinely, pulmonary arteriography and intracardiac echocardiography (ICE) have shown to be helpful in location of the valve. In our study 62.5% of PA‐VAs originated from the APC and RPC. This finding is consistent with the study by Zang et al where most of the PA‐VAs were from APC and RPC.^8^ In contrast, another study reported, 97.87% of PA‐VA originated from LPC.^12^

### 4.2 Electrocardiographic features

Pulmonary artery‐VAs showed wider QRS duration and higher incidence of lead I positivity compared to RVOT‐VAs. All PA‐VAs had precordial transition at V3 and beyond. There is no distinct ECG findings to identify the PA‐VA from RVOT‐VAs as the RVOT and PA are contiguous structures separated only by the pulmonary valve. The pulmonary valvular cusps, namely RPC and APC attach to the free wall whereas the LPC attaches to the septum. Site of successful ablation in PA is likely to be RPC, LPC, or APC when the earliest activation in RVOT is in midposterior free wall, midposterior septal side, and anterior free wall/septal side, respectively.^12^ Amplitude of the R wave in inferior leads is expected to be greater in supra cuspal VAs due to their higher location compared to RVOT. But this finding was not observed uniformly in this as well as other studies.^7^, ^13^ However, Sekiguchi et al showed R wave amplitude in inferior leads were higher in PA‐VA compared to RVOT.^9^ This may be attributed to the fact that 92% of PA‐VAs were located above the LPC. When VAs originates from LPC, there is a simultaneous activation of right and left ventricle resulting in taller R wave and narrower QRS. Since 75% of PA‐VA in our study were from APC and RPC, which usually exit into the free wall, the QRS duration was wider.

### 4.3 Electrophysiological characteristics of PA‐VA

Bipolar mapping of the VAs in the PA revealed multicomponent electrograms in 50% consisting of sharp potential followed by a dull electrogram without an intervening isoelectric segment. While the rest 50% showed a discrete potential with isoelectric line. In these patients, the site of discrete prepotential aided in the identification of the successful ablation site. The sharp potential represents near field activation of the muscular remnants in PA preceding the dull far field ventricular activation electrogram from the RVOT. This relationship reversed in sinus rhythm. Unique feature of the bipolar signal in this study was identification of a discrete prepotential at the arrhythmic focus. This discrete prepotential was first described by Miyazaki et al but subsequent studies did not show this finding in PA‐VAs.^14^ This is similar to the prepotential found in PVC originating from the aortic cusps. Hachiya et al hypothesized the origin of LVOT prepotential were from the dead‐end tract at the top of the ventricular septum.^15^ Since both out flow tracts overlap each other, prepotential in PA‐VAs may be due to a similar mechanism.

We also employed pace‐mapping in all patients at site of earliest activation. In PA‐VAs group, good pace‐map (score ≥20) was obtained in 87.5%. This high percentage was possible due to improved catheter contact by reverse U method. Due to the smaller muscle mass in the PA, higher output was required to capture the myocardium (10 mA) in this study. On the contrary, Sekiguchi et al was able to perform satisfactory pace mapping only in 37% of patients with the conventional method.^9^ A change in QRS morphology after initial ablation in RVOT did not occur in our study because when the early suppression of arrhythmia was not observed within 10 ‐15 seconds of energy delivery, we immediately switched over to PA for further mapping. In summary, activation map with early signals consisting of sharp potential preceding the ventricular electrogram along with good pace‐map identify the foci in the PA.

In this study, both acute and long term procedural success are better compared to studies using RVOT ablation without PA mapping. In a multicenter outcome study of RFCA of PVCs, recurrence occurred in 18% of RVOT patients during a mean follow‐up of 20.2 ± 21.7 months.^16^ In that study, all PVCs were ablated only in the RVOT. This high failure rate can be explained by the nature of muscular connections between the RVOT and PA, which is characterized by a triangular muscular sleeve, with broader base in RVOT requiring more RFCA lesions over a wide area compared to tapered distal end in PA which has a smaller area.

### 4.4 Limitations of the study

This is a single‐center retrospective study with a small sample size. Smaller sample size prevented a comparative study of ECG characteristics among the three PA cuspal positions. Our approach of switching over to PA instead of upfront mapping of PA may not provide a true PA‐VAs prevalence. ICE which is more precise in conforming and localizing the catheter tip was not utilized in this study.

## 5 CONCLUSION

In this study, approximately 25% of RVOT‐VAs originated from the PA. Ablation by inverted U method is safe and efficacious with good long‐term results. Discrete prepotential, good pace‐map, and early activation time help in identification of the PA–VA foci.

## CONFLICT OF INTEREST

The authors declare no conflict of interests for this article.
